# Recent Advance in Small Molecules Targeting RhlR of *Pseudomonas* *aeruginosa*

**DOI:** 10.3390/antibiotics11020274

**Published:** 2022-02-19

**Authors:** Taehyeong Lim, So-Young Ham, SangJin Nam, Myoungsun Kim, Ki Yong Lee, Hee-Deung Park, Youngjoo Byun

**Affiliations:** 1 College of Pharmacy, Korea University, Sejong 30019, Korea; ipec44@korea.ac.kr (T.L.); nj16@naver.com (S.N.); kimms624@korea.ac.kr (M.K.); kylee11@korea.ac.kr (K.Y.L.); 2 School of Civil, Environmental and Architectural Engineering, Korea University, Seoul 02841, Korea; syham1225@gmail.com (S.-Y.H.); heedeung@korea.ac.kr (H.-D.P.); 3 KU-KIST Graduate School of Converging Science and Technology, Korea University, Seoul 02841, Korea; 4 Biomedical Research Center, Korea University Guro Hospital, Seoul 08308, Korea

**Keywords:** *Pseudomonas* *aeruginosa*, RhlR, biofilm

## Abstract

*Pseudomonas* *aeruginosa* (*P*. *aeruginosa*) is an opportunistic gram-negative pathogen that can cause various infections, particularly in patients with compromised host defenses. *P*. *aeruginosa* forms biofilms and produces virulence factors through quorum sensing (QS) network, resulting in resistance to antibiotics. RhlI/RhlR, one of key QS systems in *P*. *aeruginosa*, is considered an attractive target for inhibiting biofilm formation and attenuating virulence factors. Several recent studies examined small molecules targeting the RhlI/RhlR system and their in vitro and in vivo biological activities. In this review, RhlR-targeted modulators, including agonists and antagonists, are discussed with particular focus on structure-activity relationship studies and outlook for next-generation anti-biofilm agents.

## 1. Introduction

**Figure 1:**
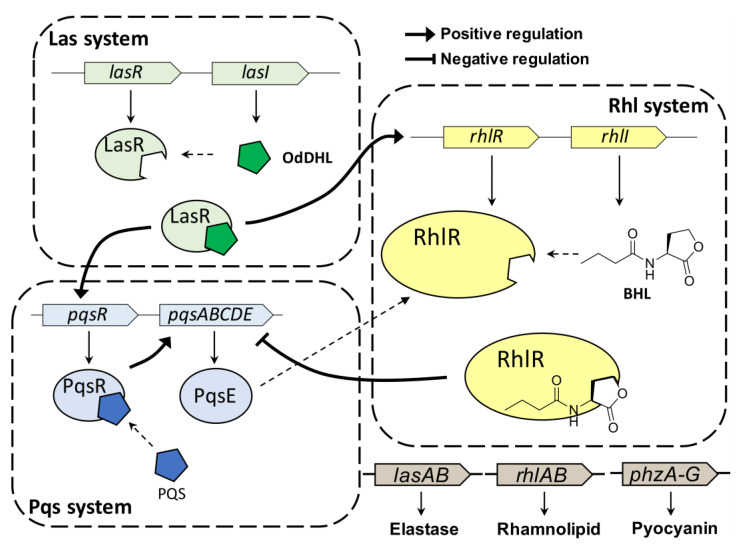
QS hierarchy in *P*. *aeruginosa*. RhlR is controlled in BHL-dependent or BHL-independent manner.

*Pseudomonas aeruginosa* (*P*. *aeruginosa*) is an opportunistic human pathogen that can cause various infections, particularly in patients with compromised host defenses [1]. *P*. *aeruginosa* is one of the so-called “ESKAPE” panel pathogens (i.e., *Enterococcus facium*, *Staphylococcus aureus*, *Klebsiella pneumonia*, *Acinetobacter baumannii*, *Pseudomonas aeruginosa* and *Enterobacter species*) [2]. *P*. *aeruginosa* can form biofilms and produce virulence factors through quorum sensing (QS), resulting in resistance to antibiotics and to the host immune response [3]. QS is a cell–cell communication process that allows bacteria to share information on bacterial population density and behave as a community to respond to changes in their environment [4]. This intercellular communication process is controlled by interactions between autoinducers and their cognate receptors. *P. aeruginosa* has three major cellular communication QS systems (Figure 1), (i.e., LasI/LasR, RhlI/RhlR, and PQS/PqsR), which are tightly interconnected [5]. This QS network of *P*. *aeruginosa* affects the production of virulence factors, biofilm formation, and modulation of host immune responses.

*P*. *aeruginosa* uses *N*-acyl-L-homoserine lactones (AHLs) as QS auto-inducers, similar to other gram-negative bacteria. AHLs are typically produced by LuxI-type synthases and are recognized by the cytoplasmic LuxR-type receptor [6]. *P*. *aeruginosa* produces *N*-(3-oxo-dodecanoyl)-L-homoserine lactone (OdDHL) and *N*-butyryl-L-homoserine lactone (BHL) for the LasI/LasR and RhlI/RhlR QS systems, respectively [7]. Once the bacteria reach a certain population density threshold, AHLs bind their cognate receptor protein, thereby affecting gene expression through transcriptional activation [8]. In addition to the LasI/LasR and RhlI/RhlR QS systems, the 2-heptyl-3-hydroxy-4(1H)-quinolone (*Pseudomonas* quinolone signal, PQS) circuit is the third system regulated by PqsR, which relies on PQS. Recently, many studies reported the interaction between Rhl and Pqs systems [9,10,11]. RhlR negatively regulates the expression of *pqsABCDE* operon independently of PQS production. Additionally, PqsE, the final gene in the operon, activates RhlR. These three QS systems are controlled in a hierarchical fashion in *P. aeruginosa*, contributing to fighting of them [5].

However, QS has challenges of selectivity, virulence reduction, and lack of resistance against QS inhibitors to reach the treatment of people [12]. The disruption of QS signals affects indirectly or directly the disturbance between microflora QS activity and other QS-mimics dependent host-microbiota signaling [13,14]. Furthermore, *P. aeruginosa* promotes the development of isolates with an increased survival ability against QS inhibitor and changes their metabolism for developing resistance of QS inhibitor [15,16]. Despite the limitations of QS inhibitors, modulating the QS network between auto-inducers and their cognate receptors is still considered a promising strategy for attenuating virulence factors of *P*. *aeruginosa* [17].

The LasI/LasR system is considered a primary target and has been studied extensively because it is located at the top of the *P*. *aeruginosa* QS hierarchy [18,19,20]. Although the RhlI/RhlR system also plays an important role in the QS process of *P*. *aeruginosa* by utilizing BHL as an autoinducer, only few studies examined RhlR-targeted modulators based on the chemical structure of BHL [21,22]. Increasing research on RhlR-targeted modulators have provided the evidence that the RhlI/RhlR system play unique roles in the QS pathway of *P*. *aeruginosa*.

According to the recent report, LasR-mutants occur frequently among environmental and clinical isolates are increasing [23,24]. There are increasing evidence that such LasR-mutants have growth advantage over the wild-type for nutrient available in the infected lungs [24,25]. In addition, many clinically isolated LasR-mutants are still able to produce RhlR-dependent transcription factors [25,26]. More than half of the LasR-mutants retain LasR-independent RhlR activity [27]. Overall, it became clear that LasR-mutants are common in a variety of chronic infections and highlight the importance of RhlR role in chronic *P*. *aeruginosa* infections [28]. Furthermore, LasR becomes dispensable in *P. aeruginosa* when it is cultured in a low phosphate medium, suggesting RhlR is the head of the QS hierarchy under phosphate-limiting conditions [29]. Therefore, small molecule modulators targeting RhlR can be developed as novel therapeutic agents in the control of *P*. *aeruginosa* chronic infections.

This review describes structure-activity relationship (SAR) studies of RhlR-targeted agonists and antagonists and discusses RhlR-targeted drug opportunities as anti-biofilm agents. The structural relationship of RhlR-targeted modulators (agonists and antagonists) was analyzed by classifying tail, middle, and head sections, inducing detailed SAR studies compared to the previous RhlR studies [30,31]. Furthermore, the importance of developing RhlR modulators for treating patients infected with *P. aeruginosa* was emphasized under LasR-mutants and phosphate-limiting conditions.

## 2. RhlR-Targeted Modulators

### 2.1. RhlR-Targeted Agonists

**Figure 2:**
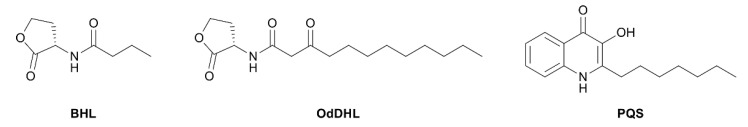
Chemical structure of auto-inducers BHL, OdDHL and PQS.

Research on RhlR-targeted modulators has mainly focused on RhlR agonists. The structural scaffold of initial RhlR agonists was based on BHL, a natural auto-inducer of RhlR (Figure 2). BHL possesses an *n*-butanoyl group at the tail region and a homoserine lactone moiety at the head region with an amide linkage. BHL further comprises a shorter alkyl chain than OdDHL, an auto-inducer responsive to LasR, and PQS, an auto-inducer responsive to PqsR (Figure 2).

Structural modification of BHL-based RhlR agonists has been implemented as follows: replacement of the homoserine lactone ring, variation of the alkyl chain, bioisosterism of the amide linkage, and absolute stereochemistry at the chiral center.

Blackwell and co-workers synthesized various BHL analogs and evaluated their EC_50_ (the effective concentration of a compound that gives half-maximal response) values using the RhlR reporter systems of *E*. *coli* and *P*. *aeruginosa* [32]. A dose-response curve of the most active RhlR agonists was analyzed to determine their EC_50_ values. They analyzed the effect of a branched alkyl chain or a cycloalkane ring at the tail region on RhlR activation. In addition, they evaluated the importance of the homoserine lactone ring at the head region regarding RhlR agonism. The BHL analog (**1**) with the isovaleryl group at the tail region showed stronger RhlR agonism with an EC_50_ value of 1.42 μM, compared to BHL (EC_50_ = 8.08 μM) in the *P*. *aeruginosa* reporter system (Table 1). Compound **2** with a cyclopropylacetyl group also showed strong RhlR agonism with an EC_50_ value of 2.76 μM in the *E*. *coli* reporter system. Introduction of cycloalkane ring such as cyclobutane (**3**, EC_50_ = 1.41 μM) or cyclopentane (**4**, EC_50_ = 1.22 μM) instead of the lactone ring enhanced RhlR agonistic properties compared to BHL in *E. coli* reporter system. In addition, replacement of the homoserine lactone ring with the homocysteine thiolactone ring (**5**, EC_50_ = 3.82 μM) slightly increased RhlR agonism in *E. coli* reporter. Furthermore, the thiolactone analogs with isovaleryl (**6**, EC_50_ = 2.58 μM) or cyclobutanyl (**7**, EC_50_ = 1.65 μM) were as potent as the corresponding the lactone analogs (**1** and **3**) in the *P*. *aeruginosa* RhlR reporter assay system [33], implying that the thiolactone ring can be a surrogate of the lactone ring. In particular, the thiolactone analog **6** displayed the strongest RhlR agonism with an EC_50_ value of 0.46 μM in the *E*. *coli* RhlR reporter assay system. When the lactone ring of BHL was replaced by cyclopentanone (**8–10**), RhlR activities were markedly decreased, compared to the corresponding lactone or thiolactone analogs in *E. coli* and *P. aeruginosa* reporter systems [20]. In addition, the reduction of the ketone to alcohol precluded the RhlR agonism, suggesting that the carbonyl group in the ring at the head region is essential for RhlR agonism between two different reporters [21]. Ring expansion from cyclopentanone (**8**) to cyclohexanone maintained RhlR agonism activity in both systems [21].

In case of homoserine lactone analogs, the extension of butyl chain to pentenyl chains at the tail region (**11** and **12**) slightly enhanced RhlR agonism in *E. coli*. In addition, the methyl branching in the propionyl (**13**) or butyryl (**14**) at the tail region showed increased RhlR agonistic activity compared to BHL in *E*. *coli* reporter system.

Blackwell et al. conducted comprehensive structure-activity relationship studies of BHL-based RhlR agonists by focusing on the tail region while retaining the homoserine lactone ring in the head region [34]. They introduced the substituted phenylacetyl, the substituted phenylpropionyl group at the tail region, and evaluated RhlR agonism by *E. coli* as summarized in Table 2.

Phenylacetyl analogs (**16–21**) substituted with electron-withdrawing substituents (-Cl, -I, and -CN) or electron-donating groups (-CH_3_, -OCH_3_, and -SCH_3_) at the *meta*-position displayed stronger RhlR agonism than compound **15** with no substituent. The electronic effect of the substituent at the *m*-position had little influence on RhlR activation. In contrast, the position of the substituent significantly affected RhlR activity, making the *meta*-substituents more potent than *para*- or *ortho*-substituents in this series. Among *m*-substituted phenylacetyl analogs, compound **20** with a -CN group at the *m*-position was most potent, with an EC_50_ value of 1.7 μM in the *E*. *coli* reporter system. However, this compound showed only approximately 70% of the maximum RhlR activity, compared to BHL. In the case of phenylpropionyl analogs, three compounds (**22–24**) displayed EC_50_ values comparable to that of BHL. However, phenylpropionyl analogs, in general, were less potent than the corresponding phenylacetyl analogs, indicating that carbon chain length in the tail region is critical for maintaining and maximizing RhlR agonism. In addition, the phenylpropionyl analogs activated LasR, PqsR, and RhlR, leading to a decrease in RhlR selectivity. Interestingly, phenylacetyl analogs substituted with the bulky group at the *para*-position turned out to be RhlR antagonists. (See Section 2.2).

Luk and co-workers reported a non-BHL RhlR agonist. Bicyclic brominated furan compound **25**, the so-called 6-bromo-4,5-dihydro-2H-cyclopenta[b]furan-2-one (5-BBF), displayed moderate RhlR agonistic activity in the PA01 system (Table 2) [35]. 5-BBF is the only compound comprising a scaffold that is not related to the homoserine lactone ring, as found in BHL analogs. However, 5-BBF was much less potent than BHL-based RhlR agonists, with an EC_50_ value of approximately 50 μM. Furthermore, this compound was not effective in inhibiting biofilm formation in *P*. *aeruginosa* and *E*. *coli*. And 5-BBF showed mild cytotoxic effects on human cells as ~76% of cells survived after 1 h of treatment.

### 2.2. RhlR-Targeted Antagonists

RhlR-targeted antagonists have also been developed based on BHL structure. Replacement of the lactone ring with a cyclopentane (**26**) or a tetrahydrofurfuryl ring (**27**) makes the parent molecule an antagonist, as summarized in Table 3. Compounds **26** and **27** showed 45% and 57% inhibition at 1 mM concentration in the presence of 10 μM BHL in the *E*. *coli* RhlR reporter assay, respectively [32]. In addition, compound **28** with a γ-lactam ring also showed weak antagonistic activity (35% inhibition). These results suggested that ring variation in the head region influences the properties of agonist or antagonist. With regard to the amide bond variation in the middle region, compound **29** with the sulfonamide linkage was a moderate RhlR antagonist with 55% inhibition. However, the compound with the ester linkage was neither an RhlR agonist nor an RhlR antagonist, implying that the hydrogen-bonding donor N-H is necessary for binding to RhlR in the BHL series [32]. The next modification in antagonists was implemented in the tail region.

Blackwell and co-workers synthesized and evaluated various phenylacetyl analogs that are bulkier than RhlR agonists with respect to molecular size (Table 4) [34]. Compounds substituted with bulky functional groups such as -I (**30**), -NO_2_ (**31**), -CH_3_ (**32**), and -CF_3_ (**33**) at the *para*-position showed strong RhlR antagonism in the *E*. *coli* RhlR reporter system, with IC_50_ (the inhibitory concentration of a compound where the response is reduced by half for dose-response curves) values ranging from 8 to 24 μM. In particular, dichloro-substituted phenylacetyl analog (**34**) exhibited the strongest RhlR antagonism with an IC_50_ value of 3.4 μM in the *E. coli* reporter system. *para*-Substituted phenoxyacetyl analogs (**35–39**) displayed strong RhlR antagonism in the *E. coli* bioassay. In particular, *para*-iodo substituted phenoxyacetyl compound **38** showed high RhlR selectivity over LasR and PqsR in *E. coli*. The antagonist effect of compound **38** was observed in the *P*. *aeruginosa* reporter system with an IC_50_ value of 23.9 μM. However, the instability of the lactone ring in culture media precluded compound **38** from further examination [33]. Based on comprehensive SAR studies, they designed and synthesized the thiolactone analog (**40**) as RhlR antagonist (Table 4). Although replacement of the homoserine lactone with the homocysteine thiolactone ring decreased RhlR antagonist activities slightly, compound **40** was a strong RhlR antagonist, with an IC_50_ values of 19.6 μM and 31.4 μM in the *E*. *coli* and *P*. *aeruginosa* reporter systems, respectively. The thiolactone ring is generally more unstable than the lactone ring because the C-S bond strength is weaker than the C-O bond. However, stability studies showed that the thiolactone compound **40** was more stable than the corresponding lactone compound **38**. 

The EC_50_ or IC_50_ values between *P. aeruginosa* and *E. coli* reporter did not often match accurately [32,34]. *P. aeruginosa* has a thicker, less permeable outer membrane, which promotes efflux pathways for small molecules to be exported both in and out of the cell more easily [36,37]. The MexAB-OprM efflux pump in *P. aeruginosa* has been shown to play a role in the transfer of many small molecules including native and non-native AHLs [38]. Therefore, it is estimated that the substrate specificity of the MexAB-OprM efflux pump and cell membrane diffusion rate could have a significant impact on the EC_50_ or IC_50_ values in *P. aeruginosa* [39,40]. However, *P. aeruginosa* would be the most useful reporter strain for evaluating the activity of BHL analogs, as this strain is RhlR’s native background [38].

Bassler and co-workers also reported that a *meta*-bromo aryl homocysteine thiolactone analog (**41**, mBTL) was a partial agonist/partial antagonist of both RhlR and LasR in the *E*. *coli* assay system [22] (Table 5). They used *E. coli* BL21 carrying plasmid pET23b containing *rhlR* and plasmid pEVS141 containing the *rhlA* promoter-driving expression of *gfp* to measure RhlR transcriptional level more directly. To determine whether analogs act as an antagonist or agonist, BHL and the analog were reacted with reporter strain in the antagonism test, whereas only analog was reacted in the agonism test. Replacement of Br with Cl in the phenyl ring retained the mixed agonism/antagonism effect and inhibition of pyocyanin production without affecting *P*. *aeruginosa* PA14 growth [22], suggesting that RhlR is well tolerated with structural modifications in the tail region. With regard to the absolute configuration of the homocysteine thiolactone ring, the (*S*)-enantiomer, a natural amino acid type, was more potent than the corresponding (*R*)-enantiomer [22]. Treatment with mBTL results in a decrease in the average height of biofilm by 64%, delaying time to clogging of microfluidic chambers. Moreover, *P. aeruginosa* rapidly killed 77% of *C. elegans* after 24 h, but when 50 μM of mBTL was treated on *C. elegans*, the killing rate decreased to 23%. mBTL also reduced the killing of human lung cells by *P. aeruginosa* and was not toxic at 100 μM.

Kato and co-workers synthesized and evaluated the effects of acyl cyclopentylamides in *P*. *aeruginosa* PAO1 [41]. They distinguished the antagonism activities of LasR and RhlR with different specific reporter strains. RhlR antagonism activity was evaluated with *P. aeruginosa* PAO1 introduced *rhlA*-*lacZ* transcriptional fusion gene by plasmid pβ01, whereas PAO1 with plasmid pβ02 carrying *lasB*-*lacZ* transcriptional fusion gene was used for the LasR antagonism. The β-galactosidase assay revealed that *N*-decanoyl cyclopentylamide (**42**) is a weak RhlR antagonist, with an IC_50_ value of 90 μM for *rhlA*-*lacZ* expression in *P*. *aeruginosa* PAO1 (Table 5). However, this compound also displayed LasR-inhibitory activity with an IC_50_ value of 80 μM for *lasB*-*lacZ* expression due to presence of a long alkyl chain group in the tail region. 250 μM of compound **42** reduced the production of elastase, rhamnolipid, and pyocyanin to 23%, 13%, and 36%, respectively [41]. And in presence of compound **42**, *P. aeruginosa* biofilm was not formed even after 1 week of cultivation.

Recently, Byun and co-workers screened RhlR antagonism of gingerol analogs with various alkyl chain lengths from 4-gingerol to 10-gingerol [42]. Compound **44** (4-gingerol) with the *n*-butyl chain in the tail region showed 31% RhlR inhibition at 100 μM in the presence of 10 μM BHL in the *E*. *coli* QS reporter strain assay (Table 6). Based on the chemical structure of 4-gingerol, they synthesized a variety of 4-gingerol analogs and evaluated RhlR antagonism. The compound structures tested in this study were not related to that of BHL. In particular, the substituted phenyl ring was utilized in the head region, instead of the homoserine lactone ring. Furthermore, the amide linkage was replaced by a simple carbonyl group. Among the diverse substituents in the phenyl ring of the head region, compound **45** with difluoro substituents at the 3- and 4-position was the most potent, leading to the replacement of 3-OCH_3_ and 4-OH substituents in 4-gingerol. Compound **45** exhibited 69% RhlR inhibition at a concentration of 100 μM. Structural optimization of compound **45** resulted in the discovery of compound **43** (Table 5), which was the most potent RhlR antagonist with 86% inhibition at 100 μM, with an IC_50_ value of 26 μM in the *E*. *coli* RhlR reporter system. The reduction of the ketone group in compound **43** to alcohol resulted in a slight decrease in RhlR antagonism. Although the absolute configuration had little effect on RhlR inhibition, the (*R*)-enantiomer (**46**) was more potent than the corresponding (*S*)-enantiomer. Molecular docking studies of compound **43** with the RhlR homology model suggested that the strong *π*-*π* stacking interaction of the 3,4-difluorophenyl ring with Tyr 71 residue, which is one of the key amino acids that interact with BHL-based RhlR modulators. Molecular docking studies of the RhlR homology model with BHL analogs using Glide software by Ravi et al. also proposed that the native auto-inducer interacts strongly with the two amino acids (Thr 57 and Tyr 71) in the active site of RhlR [43]. Moreover, compound **43** displayed strong inhibition of biofilm formation in static and dynamic settings and the reduction of virulence factor production (elastase, rhamnolipid, and pyocyanin) in *P. aeruginosa*. In addition, compound **43** did not cause toxicity to human lung epithelial cells and alleviated the infectivity of *P. aeruginosa* in *Tenebrio molitor* larvae [44].

## 3. Discussion and Conclusions

**Figure 3:**
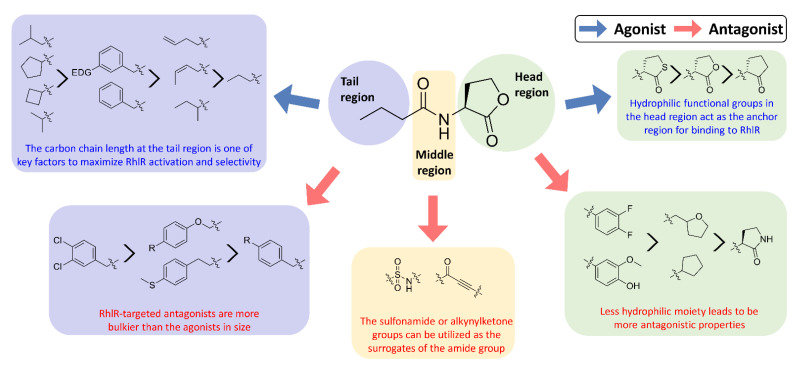
SAR summary of RhlR-targeted agonists and antagonists.

Recent SAR studies have shown the structural characteristics of RhlR-targeted agonists and antagonists. In general, receptor antagonists are more bulky in molecular size and have additional binding subpockets, compared to the corresponding agonists when they compete against the same active site of the target protein. As summarized in Figure 3, RhlR-targeted antagonists are slightly bulkier than the agonists. Homoserine lactone, homocysteine thiolactone, and cyclopentanone in the head region are commonly found in both RhlR-targeted agonists and antagonists, suggesting that a hydrophilic functional group in the head region acts as the anchor region for binding to RhlR. Replacement of the homoserine lactone with cyclopentane, tetrahydrofuran, and γ-lactam ring makes the parent molecule less hydrophilic, which leads to more antagonistic properties. In addition, introduction of the substituted phenyl ring in the head region renders the parent molecule an RhlR antagonist. In the middle region, structural modification is relatively limited compared to the head and tail regions. The sulfonamide or alkynylketone groups can be utilized as surrogates of the amide group for RhlR antagonists. In the tail region, the branched alkyls (e.g., isobutyl and isopropyl) and the cycloalkyl rings (e.g., cyclobutane and cyclopentane) were more favorable for RhlR agonism, compared with the *n*-propyl group in BHL. In the case of RhlR-targeted antagonists, the more bulky moieties including 2,4-dichlorophenylmethyl, *p*-substituted phenoxymethyl and *p*-substituted phenylmethyl are preferred in the tail region. However, there have been few reports on RhlR-targeted modulators to establish comprehensive SAR studies. Most QS inhibitors of *P*. *aeruginosa* target LasR because it is located at the top of the *P*. *aeruginosa* QS network hierarchy. From a viewpoint of drug discovery and development of RhlR-targeted modulators, X-ray crystal structures of RhlR in the presence or absence of a ligand should be determined and utilized. The lack of a RhlR 3D structure is a major obstacle to the discovery and development of novel potent and selective RhlR-targeted modulators through structure-based drug design.

*P*. *aeruginosa* is a leading cause of airway infections in patients with cystic fibrosis (CF). In isolates from CF patients with chronic *P*. *aeruginosa* infections, LasR mutations are commonly observed [24,45,46]. In these CF isolates, RhlR plays a key role in encoding virulence factors in a LasR-independent manner [28]. Dandekar et al. studied E90, a CF isolate which contains a single-base-pair deletion in *lasR* and uses RhlI/RhlR to mediate QS. RhlR produces QS-regulated virulence factors in E90 isolates, and it was the critical determinant of cytotoxicity in a 3-D lung epithelium cell model [28]. In general, the BHL/RhlR system activates the expression of genes encoding virulence factors including pyocyanin, rhamnolipid, and elastase [28,47]. However, Bassler and co-workers found that RhlR also responded in the absence of BHL and was responsible for BHL-independent transcription activities related to biofilm formation and virulence factor production [48]. The *P*. *aeruginosa* Δ*rhlI* mutant was virulent in animal infection models while the Δ*rhlR* mutant was avirulent, suggesting that BHL-independent regulation by RhlR may be more important for pathogenicity in *P*. *aeruginosa* infection [48]. The importance of RhlR was also supported by Ferrandon et al. who found that *rhlI* mutants were more virulent than *rhlR* mutants both in fly and in nematode intestinal infection models [49]. Furthermore, other studies show that in addition to atypical strains, the QS system can be flexible under certain environmental conditions, particularly for phosphate limitation [50,51]. When *P. aeruginosa* establishes infections, the phosphate level of patients undergoing chemotherapy or surgery is 0.03 mM, which is extremely low compared to healthy people (1.25 mM) [52]. Under phosphate-limiting conditions, the production of virulence factors in *P. aeruginosa* was increased [53,54]. Moreover, Soto-Aceves et al. discovered that LasR is indispensable to activate QS response, which suggested that RhlR is at the top of the QS hierarchy [29]. This phenomenon is supported by the fact that the activity of elastase, a LasR-specific virulence factor, is dependent on the Rhl system under phosphate-limiting conditions.

Overall, RhlR is an important QS transcription factor and may be a potential target for the treatment of *P*. *aeruginosa* infections, particularly in CF patients. Therefore, small molecule modulators targeting RhlR may be developed as novel antimicrobial agents for the control of *P*. *aeruginosa* infections. RhlR X-ray crystal structure, structural optimization of current RhlR-targeted agonists/antagonists, comprehensive in vivo efficacy studies, and synergistic effects with antibiotics will help develop and optimize the next generation of RhlR-targeted modulators. These efforts will be of use to promote preclinical and clinical studies, which may produce a proof-of-concept of targeting RhlR as a new therapeutic strategy to control *P*. *aeruginosa* infections.

## Figures and Tables

**Table 1:** RhlR-targeted agonists based on BHL.

Entry	Structure	EC_50_ in *E*. *coli* RhlR Reporter (μM)	EC_50_ in *P*. *aeruginosa* RhlR Reporter (μM)
**BHL**	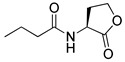	8.95	8.08
**1**	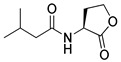	1.02	1.42
**2**	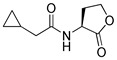	2.76	
**3**	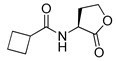	1.78	1.41
**4**	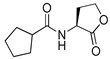	1.58	1.22
**5**	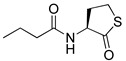	4.87	3.82
**6**	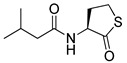	0.46	2.58
**7**	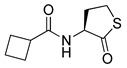	1.72	1.65
**8**	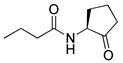	27.4	14.3
**9**	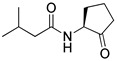	7.58	11.2
**10**	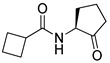	5.94	7.35
**11**	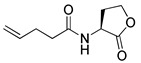	7.93	
**12**	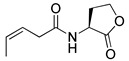	6.93	
**13**	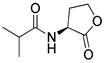	4.89	
**14**	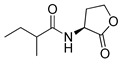	7.77	

EC_50_: the effective concentration of a compound that gives half-maximal response.

**Table 2:** RhlR-targeted agonists with variation of tail region.

Entry	Structure	EC_50_ in *E*. *coli* RhlR Reporter (μM)
**15**	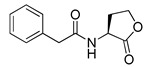	14.7
**16**	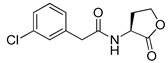	5.5
**17**	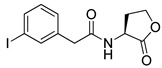	5.8
**18**	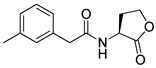	2.0
**19**	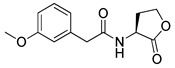	4.7
**20**	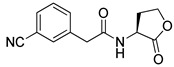	1.7
**21**	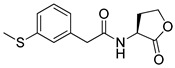	6.6
**22**	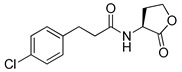	6.6
**23**	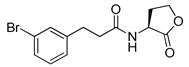	11.1
**24**	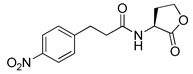	27.1
**25** **(5-BBF)**	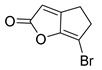	~50 (GFP expression by PA01)

**Table 3:** RhlR-targeted antagonists.

Entry	Structure	% Inhibition at 1 mM in the Presence of 10 μM BHL in *E. coli* RhlR Reporter
**26**	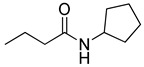	45
**27**	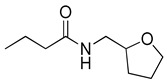	57
**28**	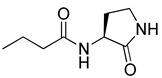	35
**29**	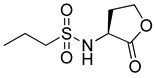	55

**Table 4:** Phenylacetyl or phenoxyacetyl analogs as RhlR antagonists.

Entry	Structure	IC_50_ in *E*. *coli* RhlR Reporter (μM)	IC_50_ in *P*. *aeruginosa* RhlR Reporter (μM)
**30**	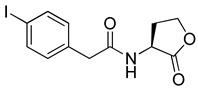	8.1	
**31**	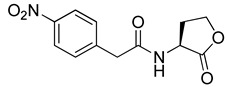	17.9	
**32**	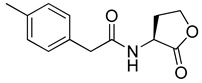	20.0	
**33**	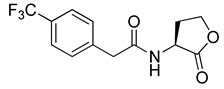	24.4	
**34**	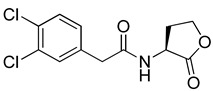	3.4	
**35**	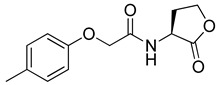	10.7	
**36**	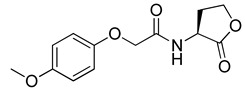	12.0	
**37**	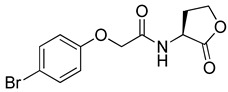	5.9	
**38**	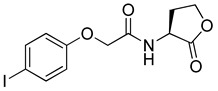	17.3	23.9
**39**	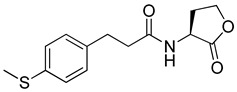	21.8	
**40**	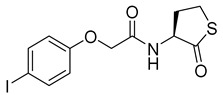	19.6	31.4

IC_50_: half maximal inhibitory concentration.

**Table 5:** Non-BHL RhlR antagonists.

Entry	Structure	IC_50_ (μM)	Reporter System
**41** **(mBTL)**	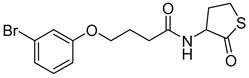		
**42** **(C10-CPA)**	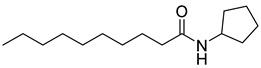	90	*P. aeruginosa* RhlR reporter
**43**	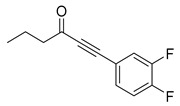	26	*E. coli* RhlR reporter

**Table 6:** Gingerol-based RhlR antagonists.

Entry	Structure	% Inhibition in *E*. *coli* RhlR Reporter
**44** **(4-Gingerol)**	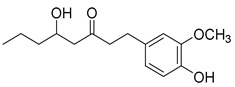	31
**45**	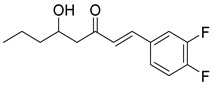	69
**46**	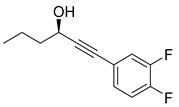	78
